# Incidence of advanced colorectal cancer in Germany: comparing claims data and cancer registry data

**DOI:** 10.1186/s12874-019-0784-y

**Published:** 2019-07-08

**Authors:** Katja Anita Oppelt, Sabine Luttmann, Klaus Kraywinkel, Ulrike Haug

**Affiliations:** 1 Department of Clinical Epidemiology, Leibniz Institute for Prevention Research and Epidemiology – BIPS, Achterstraße 30, 28359 Bremen, Germany; 2 Bremen Cancer Registry, Leibniz Institute for Prevention Research and Epidemiology – BIPS, Bremen, Germany; 3 Department for Epidemiology and Health Reporting, German Centre for Cancer Registry Data, Robert Koch-Institute, Berlin, Germany; 4 Faculty of Human and Health Sciences, University of Bremen, Bremen, Germany

**Keywords:** Colorectal neoplasms, neoplasm staging, neoplasm metastasis, administrative claims, healthcare

## Abstract

**Background:**

Incidence rates of advanced cancer stages are important, e.g., for monitoring cancer screening programs. However, information from cancer registries on tumor stage is often incomplete. Exemplified by colorectal cancer (CRC), we explored the potential of German claims data to estimate incidence rates of advanced cancer stages.

**Methods:**

We used claims data of the German Pharmacoepidemiological Research Database (GePaRD; information on > 20 million persons) to identify incident patients with advanced CRC based on ICD-10 codes for CRC and secondary malignant neoplasms. We calculated annual age-standardized incidence rates (ASIRs) of advanced CRC per 100,000 for the years 2008–2015 stratified by the presence of affected lymph nodes only (C77) vs. distant metastases (C78-C79) and compared them to ASIRs determined using data (2008–2014) from the German Centre for Cancer Registry Data (ZfKD).

**Results:**

In GePaRD, the ASIRs of advanced CRC per 100,000 in 2014 were 21.5 among men and 14.9 among women. Compared to ZfKD data the ASIR in GePaRD was 2.58 lower in men and 0.27 higher in women (per 100,000) in 2014. Stratification by presence of distant metastases showed divergent patterns: the ASIRs regarding distant metastases were ~ 50% (women) and ~ 30% (men) higher, and the ASIRs regarding affected lymph nodes only were ~ 40% lower in GePaRD as compared to ZfKD.

**Conclusion:**

While ASIRs of advanced CRCs overall agreed well between claims and cancer registry data in 2014, the analyses stratified by presence of distant metastases showed differences. Cancer registries might underestimate ASIRs of CRCs with distant metastases.

**Electronic supplementary material:**

The online version of this article (10.1186/s12874-019-0784-y) contains supplementary material, which is available to authorized users.

## Background

Cancer stage at diagnosis is an essential determinant of cancer survival [1–5]. While the involvement of regional lymph nodes is already disadvantageous, patients with distant metastases have the least favorable prognosis. For example, regarding the four most common cancers (i.e. breast, lung, colorectal, and prostate cancer), 5-year relative survival of patients with distant metastases ranged between of 5 to 30% as compared to 56 to 100% for localized stages [6]. The rate of cancers diagnosed with distant metastases is thus an important parameter to monitor the cancer burden.

Whereas cancer registries typically show a high level of completeness regarding cancer incidence, information on the spread to regional lymph nodes or the presence of distant metastases is often less complete [1, 2, 7, 8]. In particular, the recording of data on distant metastasis is problematic for two reasons. First, the diagnostic procedures may not be completed at the time when the cancer is reported to the cancer registry. Second, a certain proportion of cancers may be reported to the cancer registry by pathologists only who examine the tumor tissue but do not have information on distant metastases. Claims databases are an increasingly important data source in oncology to address research questions where primary or registry data are limited [9–12]. It needs to be explored whether they also bear potential to monitor the rate of advanced colorectal cancer (CRC) stages, particularly those with distant metastases. For some claims databases, algorithms to determine cancer stage have been developed, mainly for breast cancer but partly also for other cancer sites [9, 11–15]. German claims data have not been explored in this regard so far. While the potential of claims data to identify advanced cancers is of general interest from a methodological point of view, monitoring the rate of advanced CRC is of particular interest in Germany due to recent developments in CRC screening [16]. We therefore aimed to explore the potential of determining the incidence rates of advanced CRC based on German claims data and to compare them to rates determined based on cancer registry data, including trends over time.

## Methods

### Data sources

We used the German Pharmacoepidemiological Research Database (GePaRD) for this study. The database is described in detail elsewhere [17]. GePaRD is based on claims data from four statutory health insurance providers in Germany and currently includes information on more than 20 million persons who have been insured with one of the participating providers since 2004 or later. In addition to demographic data, GePaRD contains information on drug dispensations, outpatient and inpatient services and diagnoses. Per data year, there is information on approximately 17% of the general population and all geographical regions of Germany are represented.

In GePaRD, diagnosis codes are registered according to the International Statistical Classification of Diseases and Related Health Problems, 10th revision, German Modification (ICD-10 GM). For inpatient diagnosis codes, the exact date is available in German claims data, while outpatient diagnosis codes are only available on a quarterly basis. With respect to the inpatient setting, we considered main and secondary hospital discharge diagnoses, but not admission diagnoses. In the outpatient setting, the additional coding of diagnostic certainty is mandatory in Germany. This coding differentiates between “confirmed”, “suspected”, “status post” and “excluded” diagnoses. For the inclusion of incident CRCs, we only considered diagnoses from the outpatient setting coded as “confirmed”. For the exclusion of prevalent CRCs both “confirmed” and “status post” diagnoses were considered as described below.

As additional data source we used data from the German Centre for Cancer Registry Data (Zentrum für Krebsregisterdaten, ZfKD). Data from ZfKD was available for the years 2008 to 2014. The ZfKD receives data from the population-based cancer registries collecting data in each German federal state [18]. The ZfKD estimates the completeness of reported cancer cases based on the method recommended by the International Agency for Research of Cancer (IARC) for each of these registries, using cancer site specific mortality/incidence ratios of established registries as a reference [18, 19].

### Analyses of GePaRD data

To identify patients diagnosed with advanced CRC and determine the annual rate, we first needed to identify patients with an incident CRC diagnosis in a particular calendar year. For this first step, we included all patients with an in- or outpatient diagnosis code of CRC (C18-C20) in the respective year. We defined the date of the first CRC code in this year as cohort entry. We only included patients with an additional (in- or outpatient) diagnosis code for CRC in the same or in the two following quarters (i.e. within up to 6–9 months) after cohort entry to confirm the initial diagnosis. To restrict the sample to incident CRCs, we excluded patients with a code for CRC (“confirmed” or “status post”) during a preobservation period of 4 years before cohort entry. This required excluding patients whose insurance period was less than 4 years. Further, we excluded patients with an interruption of insurance of more than 15 days and patients below 5 years of age. The procedure regarding the preobservation period and the confirmatory diagnosis is in line with a previously developed algorithm to identify incident CRCs in German claims data [20].

In the second step, we considered information regarding stage at diagnosis of the incident CRC cases. Specifically, we considered codes for lymph node involvement and metastases (C77-C79) documented in the quarter of cohort entry or in the following quarter (i.e. within 3–6 months after cohort entry). We categorized patients with one in- or outpatient diagnosis code of lymph node involvement (C77) or metastases (C78-C79) as patients with advanced CRC. We also considered these patients stratified by stage, i.e. those with affected lymph nodes only (C77), corresponding to UICC III, vs. those with distant metastases (C78-C79), corresponding to UICC IV. We conducted sensitivity analyses with varying periods for considering C78-C79 diagnoses (i.e. up to 0–3 months and 6–9 months after cohort entry instead of up to 3–6 months). Furthermore, we determined the proportion of patients classified as UICC IV for whom at least one C78–79 diagnosis code was recorded as inpatient discharge diagnosis. The codes used in the analyses are listed in Additional file 1.

To roughly assess whether using only diagnostic codes leads to a substantial underestimation of the number of advanced CRCs, we considered patients not classified as advanced CRC and assessed whether they received a medical therapy that is typically only administered in advanced CRC such as Bevacizumab (see Additional file 1). For this analysis, we used the subsample of CRC patients of one SHI that provides detailed information on cancer therapy in the in- and outpatient setting. The remaining SHIs do not transfer data on specific agents included in chemotherapies in the outpatient setting.

To estimate the incidence rate of advanced CRC per year, we used the number of incident CRC cases classified as “advanced” for the respective year in the nominator. As denominator, we used the number of all individuals in GePaRD of the respective year, excluding those who were not continuously insured during a look-back period of at least 4 years. This exclusion criterion ensured comparability between the nominator and the denominator given that inclusion of CRC patients also required a look-back period of 4 years. We first determined the crude rates and then calculated age-standardized incidence rates (ASIRs) of advanced CRCs per 100,000 persons [21]. ASIRs were calculated for each calendar year (2008–2015) and stratified by sex. All ASIRs presented in this paper are calculated according to the old European Standard Population [22].

### Analyses of ZfKD data

We compared our results based on GePaRD to the incidence rate of advanced CRC determined based on data of the ZfKD. We included only data from federal state cancer registries showing an estimated level of completeness of 95% or more with respect to the incidence of CRC during the whole observation period. This resulted in the inclusion of seven federal state cancer registries covering about 32 million inhabitants of Germany (~ 39% of the general population). To categorize incident CRCs according to stage analogously to the approach applied to the GePaRD, we used information on the TNM status as far as it was available in the ZfKD data. The respective algorithm is described in Additional file 2. For each year we determined the proportion of CRCs that was not classifiable into these categories due to missing information on the N- or M-status. When calculating rates, we used the number of incident CRCs classified as “advanced” as nominator and the number of inhabitants of the federal states included in the analysis as denominator [23, 24]. Analogously to the analysis based on GePaRD, we calculated ASIRs (also standardized according to the old European Standard Population) of advanced CRCs per 100,000 persons for each calendar year (2008–2014) and stratified by sex. We also stratified the analyses by UICC III vs. IV. We conducted additional analyses regarding the impact of different approaches how to consider missing information on the N and M status in ZfKD data (see Additional files 3 and 4).

We conducted all analyses with SAS 9.3 [25].

## Results

In Table 1, we show the characteristics of the source population and the patients identified with advanced CRC for the years 2008 and 2014. The mean number of patients with advanced CRC per year was 3081. More than half of the patients with advanced CRC were male. The mean age ranged between 68.2 and 70.0 years among female and between 67.0 and 68.6 years among male patients (Table 1). In the data from ZfKD, the mean number of patients with advanced CRC per year was 10,333 (56% male patients). The mean age among female patients ranged between 70.6 and 71.0 years and among male patients between 67.6 and 68.3 years (Table 1).

**Table 1:** Source population and advanced CRC patients identified in claims data (GePaRD) and in cancer registry data (ZfKD): Distribution of age and sex exemplified for 2008 and 2014

	GePaRD		ZfKD	
	2008	2014	2008	2014
Source population				
Overall	10,333,691^a^	11,120,604^a^	32,114,493	31,784,281
Men (%)	4,596,991 (44.5%)	5,097,709 (45.8%)	15,711,368 (48.9%)	15,563,141 (49.0%)
Women (%)	5,736,700 (55.5%)	6,022,895 (54.2%)	16,403,125 (51.1%)	16,221,140 (51.0%)
Mean age [years]	45.4	47.6	44.4	45.8
Men	44.0	46.2	43.0	44.4
Women	46.5	48.8	45.8	47.1
Advanced CRC patients^b^				
Overall	2755	3370	10,750	9534
Men (%)	1439 (52.2%)	1764 (52.3%)	5919 (55.1%)	5435 (57.0%)
Women (%)	1316 (47.8%)	1606 (47.7%)	4831 (44.9%)	4099 (43.0%)
Mean age [years]	68.1	69.2	69.0	69.4
Men	67.0	68.6	67.6	68.3
Women	69.3	70.0	70.7	70.9

^a^Persons with continuous health insurance coverage of at least 4 years

^b^In one of the health insurances providing data of about 6 million insured persons to GePaRD, the proportion of women 50 years old or older is substantially higher as compared to the general population (32.1% vs. 22.5%). This explains why the gender distribution among patients with advanced CRCs differs from the distribution reported by cancer registries

**Fig. 1:**
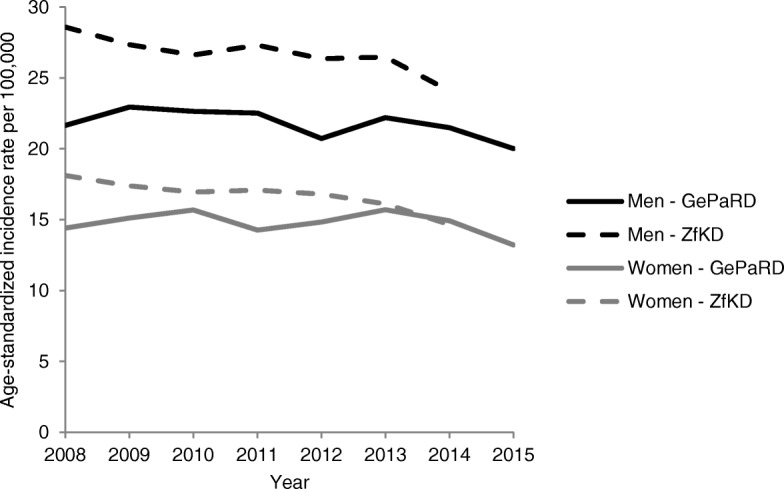
Age-standardized incidence rates (ASIRs) of advanced CRC: Comparison between claims data (GePaRD) and cancer registry data (ZfKD)

Figure 1 shows the ASIRs of advanced CRC determined based on data from GePaRD and ZfKD, respectively. According to GePaRD data, the ASIR of advanced CRC decreased from 21.6 to 20.0 in men and from 14.4 to 13.2 per 100,000 in women between 2008 and 2015. According to ZfKD data, the ASIR of advanced CRC decreased from 28.6 to 24.1 in men and from 18.1 to 14.7 per 100,000 in women between 2008 and 2014. In 2014, the most recent year for which ZfKD data were available, the ASIR in GePaRD was 2.58 lower in men and 0.27 higher in women (per 100,000) compared to ZfKD.

**Fig. 2:**
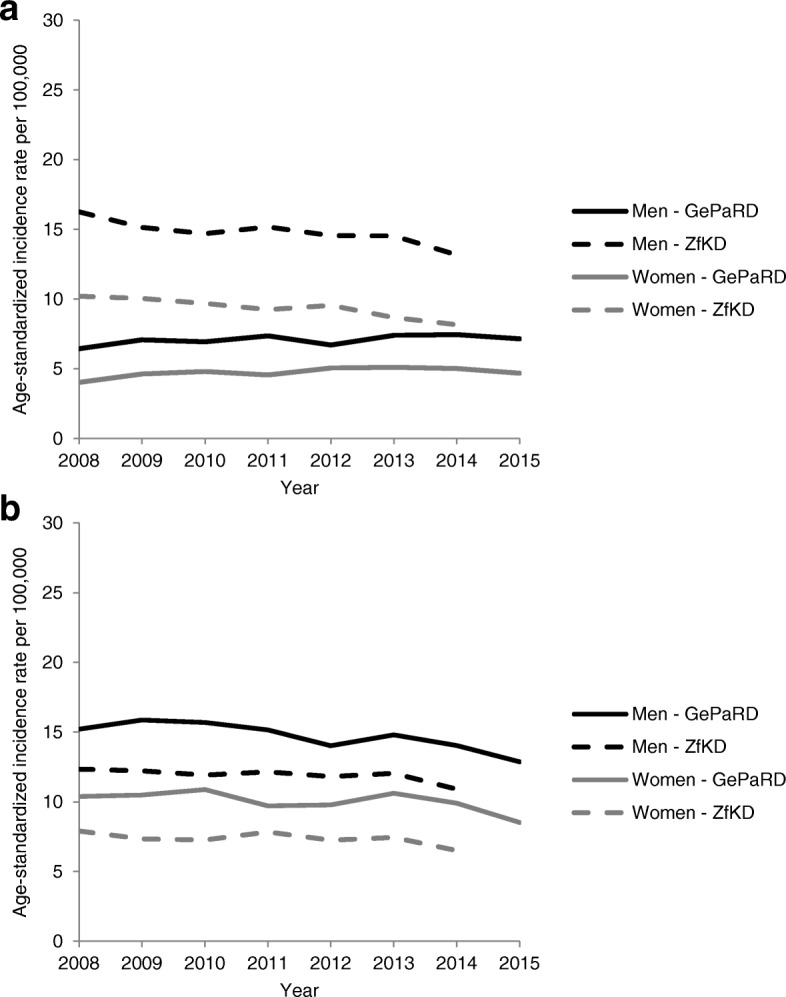
**a**. Age-standardized incidence rates (ASIRs) of advanced CRCs with affected lymph nodes only: Comparison between claims data (GePaRD) and cancer registry data (ZfKD). **b**. Age-standardized incidence rates (ASIRs) of advanced CRCs with distant metastases: Comparison between claims data (GePaRD) and cancer registry data (ZfKD)

Figure 2 shows the ASIRs of advanced CRCs stratified by the presence of affected lymph nodes only, i.e. UICC III, (Fig. 2a) vs. the presence of distant metastases, i.e. UICC IV (Fig. 2b) determined based on data from GePaRD and ZfKD, respectively. Across all years, the ASIRs of advanced CRCs with affected lymph nodes only (UICC III) was lower according to GePaRD as compared to ZfKD. In 2014, the ASIRs were 38% lower in women and 43% lower in men (Fig. 2a). An opposite pattern was observed for the ASIRs of advanced CRCs with the presence of distant metastases (UICC IV). In 2014, the ASIRs were 52% higher in women and 29% higher in men according to GePaRD as compared to ZfKD (Fig. 2b). According to GePaRD data, the ASIRs of CRC with distant metastasis (UICC IV) decreased between 2008 and 2015 from 15.2 to 12.9 in men and from 10.4 to 8.5 per 100,000 in women.

**Fig. 3:**
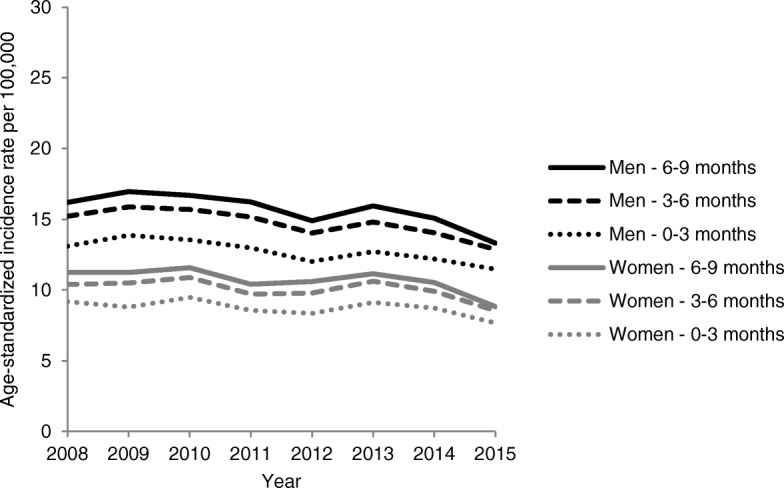
Sensitivity analyses on age-standardized incidence rates (ASIRs) of advanced CRCs with distant metastases estimated based on claims data (GePaRD): Comparison of different periods used for the consideration of C78–79 codes after cohort entry

In the sensitivity analyses using varying periods (0–3 and 6–9 months after cohort entry) for the consideration of C78-C79 diagnosis codes, the ASIR of advanced CRCs with the presence of distant metastases (UICC IV) decreased using the shorter period and increased using the longer period (Fig. 3). For 87.2% of patients classified as UICC IV in the main analysis, at least one C78–79 diagnosis code was recorded as inpatient discharge diagnosis. For two thirds of the remaining patients, there were two or more outpatient diagnosis codes (status “confirmed”) for distant metastasis. With respect to UICC III, extending the period from 3 to 6 months to 6–9 months to consider C77 diagnoses did not change the respective rates (see Additional file 5).

**Fig. 4:**
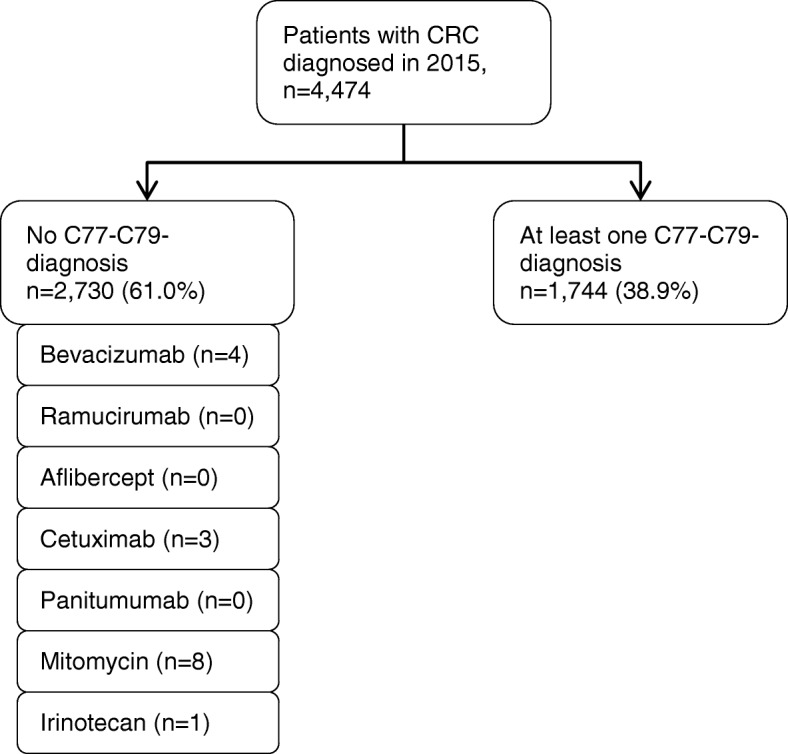
Number of patients classified as patients with non-advanced CRC who received medication typically only administered in patients with advanced CRCs (in a subsample diagnosed in 2015)

Figure 4 shows the results of our approach to assess whether the algorithm used for the GePaRD data leads to a substantial underestimation of the number of advanced CRCs. In a subsample of 4474 CRC patients for whom detailed information on administered in- and outpatient medication was available, 2730 were classified as non-advanced CRCs, i.e. no C77-C79 diagnostics codes were recorded in these patients. Of these, 16 patients (0.36%) received medication that is typically only prescribed for advanced CRC.

The results of the additional analyses regarding the impact of different approaches how to consider missing information on the N and M status in ZfKD data are described in Additional files 3 and 4. As shown in Additional file 2, the proportion of CRCs that were not classifiable as advanced or non-advanced CRCs according to the information on the N and M status in the ZfKD data decreased from 35 to 29% between 2008 and 2014. Ignoring the non-classifiable CRCs resulted in ASIRs of non-advanced CRCs that were about twice as high in GePaRD compared to ZfKD, while the ASIRs converged when we assumed that all non-classifiable CRCs were non-advanced (see Additional file 3).

The proportion of advanced CRCs that could not further be stratified by UICC stage III vs. IV decreased from 17 to 7% between 2008 and 2014 (see Additional file 2). When making the extreme assumption that all advanced CRCs that could not be stratified by UICC stage III vs. IV based on ZfKD data were exclusively UICC stage IV, the ASIRs of UICC stage IV CRCs were still slightly higher in GePaRD compared to ZfKD (see Additional file 4)b..

## Discussion

To the best of our knowledge, this is the first study that explored the potential of German claims data to estimate the incidence rate of advanced cancer stages. For advanced CRC, we found on average 10% lower rates in women and 17% lower rates in men between 2008 and 2014 when using claims data as compared to cancer registry data, with a trend towards decreasing differences in most recent data years. However, stratification by the presence or absence of distant metastases showed different patterns. The rate of CRCs with distant metastases was markedly higher (on average 26% in men and 40% in women) when determined based on claims data as compared to estimates based on cancer registry data, while for advanced CRCs with affected lymph nodes only it was the other way round. Given that cancers presenting with distant metastases show the worst prognosis, a potential underestimation of their incidence by cancer registries would be of high relevance and deserves further attention.

In the absence of a gold standard, potential limitations of both data sources need to be discussed and consideration of complementary patterns may be helpful to approach the answer regarding the true rates. In epidemiological cancer registration, a certain proportion of incident cancer cases is reported by pathologists only who do typically not have information on distant metastasis, which could lead to an underestimation of the respective rates. Furthermore, a potential delay in diagnostic procedures needs to be considered. A scenario where a certain proportion of cancers with known lymph node status is reported to cancer registries as “M0” before the procedures to diagnose distant metastases are completed could be another explanation of the patterns we observed in our study. These cancers would be assigned to the category “affected lymph nodes only” based on cancer registry data, while claims data would capture metastases diagnosed or treated after the cancer has been reported to the cancer registry. The relevance of this scenario is supported by the results of our sensitivity analyses where the rates of CRCs with distant metastases were lower when we used a follow-up period of only 3 months for the consideration of diagnosis codes in claims data. Of note, the time period of getting potential information on distant metastasis inherently differs between cancer registry and claims data. According to guidelines by the European Network of Cancer Registries (ENCR) for epidemiological cancer registration, affected lymph nodes and metastases diagnosed before start of treatment measures should be assigned to stage at diagnosis. Thus, the results of delayed diagnostic procedures may not be reported to cancer registries [26]. The recommendation of the Union for International Cancer Control (UICC), which maintains the TNM staging classification, in terms of abandoning “MX” and assigning “M0” unless there is positive evidence of metastases could be relevant regarding both explanations discussed above [27]. Accordingly, the current system and guidelines of cancer registration may lead to an underreporting of synchronous metastasis, which are typically defined as metastases diagnosed within 6 months after initial cancer diagnosis [28].

On the other hand, also the information provided by claims data needs to be questioned given that they are not primarily collected for research but for the purpose of reimbursement. However, for more than 85% of persons assigned as CRC patients with distant metastases based on claims data, an inpatient discharge diagnosis code for distant metastases was available. In Germany, inpatient discharge diagnoses are assumed to have a high validity since they are based on all information relevant to diagnosis (including laboratory tests and imaging results) during the in-hospital stay [29]. Furthermore, they are subject to regular inspections. For the vast majority of the remaining persons, there was not only one, but at least two confirmed outpatient diagnosis codes for distant metastasis. Despite potential advantages of claims data in recording distant metastasis, the overall pattern in ASIRs of advanced CRCs suggests that claims data tend to underestimate the rate of UICC stage III as compared to cancer registry data, possibly due to undercoding of lymph node involvement.

In the interpretation of the rates determined based on cancer registry data, it needs to be considered that about 30% of incident CRCs across all data years could not be classified as early or advanced cancers due to missing information on the N or M status. Still, the rates of advanced CRCs overall showed good agreement with the rates determined based on claims data. By contrast, the rates of non-advanced CRCs showed only good agreement with the rates determined based on claims data when we assumed that the CRCs with missing stage information were all non-advanced (see Additional file 3). This pattern might indirectly show that the vast majority of CRCs for which cancer registry data did not provide enough information to classify them as early or advanced were diagnosed at an early stage, but this remains speculative.

While there is no study from Germany to which we could compare our findings, there are two studies from the US that explored the potential of claims data to distinguish between early and advanced CRCs. For CRCs classified as metastatic according to an electronic medical record database, Nordstrom et al. found claims codes for distant metastases in only about 30% of these cases. However, the follow-up in this study was limited to 60 days. In addition, in was not clear whether all physicians providing oncology care to the patients were captured by the open claims system that was used for this study [9]. Chawla et al. assessed the potential of Medicare claims data for inferring stage at the time of CRC diagnosis by linking the claims to cancer registry data from the Surveillance, Epidemiology and End Results (SEER) program. They aimed to classify CRCs into the SEER historic stages (local vs. regional vs. distant) which required – unlike our approach – also information on the localization of metastases. Diagnosis codes from Medicare data showed only limited discriminatory power in this regard, with the misclassification being most pronounced in older CRC patients and in those residing in lower income areas. Overall, classification based on Medicare diagnosis codes underestimated the proportion of regional or distant cancer stages, which differs from our findings based on German claims data [12]. This emphasizes that it is not possible to draw generalized conclusion for claims databases from different countries due to differences between health systems and reimbursement policies influencing the availability and validity of codes.

Our claims data analyses showed an overall decrease in the rate of CRCs with distant metastases between 2008 and 2015 by 15% in men and 18% in women. This decreasing trend was also observed in the most recent data years. Given the time-lagged association between incidence of these CRCs and CRC mortality, the pattern suggests that the current trend of a decreasing CRC mortality in Germany will continue over the next years [30]. This decline may have several reasons but it is considered likely that the introduction of screening colonoscopy in 2002 in Germany has contributed to this trend.

We recognize both strength and limitations to our study. The claims database allowed to estimate rates based on a large sample size and to assess trends over time. Due to the long follow-up we could use look-back periods of 4 years which is advantageous in terms of reliably distinguishing incident from prevalent cancers [20]. We could not directly link claims and cancer registry data, but compared the rates indirectly and conducted thorough sensitivity analyses to ensure a careful interpretation of the findings. Since the claims data only provide diagnosis codes according to ICD-10, we could not differentiate between affected regional vs. distant lymph nodes and considered them all as regional. This leads to a potential misclassification given that the TNM system assigns affected distant lymph nodes to the M status. However, if this misclassification could be avoided the GePaRD-based rates of CRCs with distant metastasis would be even higher and the difference to the cancer registry-based rates would thus be even larger than reported in our study. It should also be noted that the codes C77-C79 do not carry the information about the primary tumor. In case of multiple cancer, the distant metastasis and affected lymph nodes might be caused by a different type of cancer.

In the interpretation of our study, it should also taken into account that we did not expect perfect agreement between the rates determined based on cancer registry versus claims data given that the study population underlying the claims data may not be fully representative of the general population in Germany. In this context, the differential agreement by gender requires further exploration. Overall, our study was not intended to question the value of population-based cancer registration, especially in view of the full population coverage and all the information on cancers (e.g. on histology, tumor size, and grading) that is not available in claims data. However, determining the rate of advanced cancer stages based on claims data could complement cancer registration, e.g. by allowing to investigate potential determinants of advanced cancer stages (co-morbidity, etc.).

## Conclusions

In conclusion, our study provides important insights into the potential of German claims data to estimate the incidence rates of advanced cancers. While ASIRs of advanced CRCs in recent years overall agreed well between claims and cancer registry data, within the group of advanced CRCs cancer registries might underestimate ASIRs of CRCs with distant metastases when lymph node involvement is present. This requires further consideration given that cancers presenting with distant metastases show the poorest survival. Amongst others, their misclassification would bias stage-specific survival estimates and underestimating the incidence of these cancers would bias projections regarding the impact of cancer screening programs on disease-specific mortality.

## Additional files

Additional file 1: Overview and description of codes used in the claims data analyses. (DOCX 15 kb)

Additional file 2: Algorithm to classify CRCs into “advanced”, “non-advanced” or “not classifiable” according to the information on the N and M status from cancer registry (ZfKD) data and description of additional analyses conducted with respect to missing information. (DOCX 15 kb)

Additional file 3: Results of additional analyses conducted to explore whether it is plausible that CRCs not classifiable into “advanced” or “non-advanced” based on cancer registry (ZfKD) data tend to be non-advanced. (DOCX 16 kb)

Additional file 4: Results of the sensitivity analyses making the extreme assumption that all advanced CRCs that could not be stratified by UICC stage III vs. IV based on cancer registry (ZfKD) data were UICC stage IV. (DOCX 27 kb)

Additional file 5: Sensitivity analyses on age-standardized incidence rates (ASIRs) of advanced CRCs with affected lymph nodes only estimated based on claims data (GePaRD): Comparison of different periods used for the consideration of C77 codes after cohort entry. (DOCX 16 kb)

## Data Availability

The datasets generated during and/or analysed during the current study are not publicly available due to data protection regulations in Germany. According to these regulations, access to the data of the German Pharmacoepidemiological Database must not be given to third parties. Furthermore, as we are not the owners of the data we are not legally entitled to grant access to the data.

## Abbreviations

ASIR: Age standardized incidence rates
ATC: Anatomical Therapeutic Chemical Classification System
CRC: Colorectal cancer
DCO: Death certificate only
EBM: Billing codes (Einheitlicher Bewertungsmaßstab)
ENCR: European Network of Cancer Registries
GePaRD: German Pharmacoepidemiological Research Database
ICD-10 GM: International Statistical Classification of Diseases and Related Health Problems, 10th revision, German Modification
OPS: German procedure classification (Operationen- und Prozedurenschlüssel)
SEER: Surveillance, Epidemiology and End Results program of the National Cancer Institute
SHI: Statutory health insurances
TNM: TNM classification of malignant tumours
UICC: Union for International Cancer Control
ZfKD: German Centre for Cancer Registry Data
