# Supplementary material for: Incidence of advanced colorectal cancer in Germany: comparing claims data and cancer registry data
Source: BMC Med Res Methodol. 2019 Jul 8;19:142. doi: 10.1186/s12874-019-0784-y (PMC6615087; doi:10.1186/s12874-019-0784-y)
Supplement: Supplementary file 1 — Overview and description of codes used in the claims data analyses. (DOCX 15 kb) [file 12874_2019_784_MOESM1_ESM.docx]

Additional file 1. Overview and description of codes used in the claims data analyses

| **Catalogue** | **Code** | **Description of the code** |
| --- | --- | --- |
| ICD-10 GM |  |  |
|  | C18.- | Malignant neoplasm of colon |
|  | C19. | Malignant neoplasm of rectosigmoid junction |
|  | C20. | Malignant neoplasm of rectum |
|  | C77.- | Secondary and unspecified malignant neoplasm of lymph nodes |
|  | C78.- | Secondary malignant neoplasm of respiratory and digestive organs |
|  | C79.- | Secondary malignant neoplasm of other and unspecified sites |
| ATC |  |  |
|  | L01DC03 | Mitomycin |
|  | L01XC06 | Cetuximab |
|  | L01XC07 | Bevacizumab |
|  | L01XC08 | Panitumumab |
|  | L01XC21 | Ramucirumab |
|  | L01XX19 | Irinotecan |
|  | L01XX44 | Aflibercept |
| OPS-6 |  |  |
|  | 6-001.3 | Irinotecan |
|  | 6-001.a | Cetuximab |
|  | 6-002.9 | Bevacizumab |
|  | 6-004.7 | Panitumumab |
|  | 6-007.2 | Aflibercept |
