# Supplementary material for: Incidence of advanced colorectal cancer in Germany: comparing claims data and cancer registry data
Source: BMC Med Res Methodol. 2019 Jul 8;19:142. doi: 10.1186/s12874-019-0784-y (PMC6615087; doi:10.1186/s12874-019-0784-y)
Supplement: Supplementary file 2 — Algorithm to classify CRCs into “advanced”, “non-advanced” or “not classifiable” according to the information on the N and M status from cancer registry (ZfKD) data and description of additional analyses conducted with respect to missing information. (DOCX 15 kb) [file 12874_2019_784_MOESM2_ESM.docx]

**Additional file 2.** Algorithm to classify CRCs into “advanced”, “non-advanced” or “not classifiable” according to the information on the N and M status from cancer registry (ZfKD) data and description of additional analyses conducted with respect to missing information

| N > 0 *OR* M > 0 | advanced |
| --- | --- |
| N=0 / missing AND M=missing | not classifiable |
| N=missing AND M=0 | not classifiable |
| All others | non-advanced |

The proportions of CRCs that were not classifiable according to this algorithm were as follows (per calendar year):

| **2008** | **2009** | **2010** | **2011** | **2012** | **2013** | **2014** |
| --- | --- | --- | --- | --- | --- | --- |
| 35.2% | 34.3% | 32.3% | 29.2% | 28.8% | 26.9% | 28.6% |

To explore whether it is plausible that the not classifiable CRCs tend to be non-advanced, we conducted additional analyses where we calculated ASIRs for non-advanced CRCs based on ZfKD data with and without consideration of not classifiable CRCs and compared these ASIRs to the ASIRs of non-advanced CRCs determined based on GePaRD. The results of these additional analyses are shown in Additional file 3.

Among the CRCs that were classified as “advanced”, those with “N=1 AND M=missing” could not further be stratified by UICC stage III vs. IV. The proportions of these advanced CRCs that could not be stratified by UICC stage III vs. IV were as follows (per calendar year; with all CRCs classified as “advanced” in the denominator):

| **2008** | **2009** | **2010** | **2011** | **2012** | **2013** | **2014** |
| --- | --- | --- | --- | --- | --- | --- |
| 17.01% | 15.81% | 11.46% | 9.07% | 9.00% | 7.28% | 7.41% |

In the base-case analyses, we assumed that these CRCs were UICC III (i.e. that no distant metastasis were present). We conducted sensitivity analyses based on the extreme assumption that these CRCs were exclusively UICC IV. The results of these sensitivity analyses are shown in Additional file 4.
