# Supplementary material for: Incidence of advanced colorectal cancer in Germany: comparing claims data and cancer registry data
Source: BMC Med Res Methodol. 2019 Jul 8;19:142. doi: 10.1186/s12874-019-0784-y (PMC6615087; doi:10.1186/s12874-019-0784-y)
Supplement: Supplementary file 3 — Results of additional analyses conducted to explore whether it is plausible that CRCs not classifiable into “advanced” or “non-advanced” based on cancer registry (ZfKD) data tend to be non-advanced. (DOCX 16 kb) [file 12874_2019_784_MOESM3_ESM.docx]

Additional file 3. Results of additional analyses conducted to explore whether it is plausible that CRCs not classifiable into “advanced” or “non-advanced” based on cancer registry (ZfKD) data tend to be non-advanced

GePaRD: Age-standardized incidence rates of non-advanced CRCs estimated based on claims data

ZfKD I: Age-standardized incidence rates of non-advanced CRCs estimated based on ZfKD data, considering only CRCs classified as “non-advanced” (see also Additional file 2)

ZfKD II: Age-standardized incidence rates of non-advanced CRCs estimated based on ZfKD data, considering CRCs classified as “non-advanced” and CRCs that were not classifiable, i.e. assuming that these CRCs were also non-advanced (see also Additional file 2)
