# Supplementary material for: Incidence of advanced colorectal cancer in Germany: comparing claims data and cancer registry data
Source: BMC Med Res Methodol. 2019 Jul 8;19:142. doi: 10.1186/s12874-019-0784-y (PMC6615087; doi:10.1186/s12874-019-0784-y)
Supplement: Supplementary file 4 — Results of the sensitivity analyses making the extreme assumption that all advanced CRCs that could not be stratified by UICC stage III vs. IV based on cancer registry (ZfKD) data were UICC stage IV. (DOCX 27 kb) [file 12874_2019_784_MOESM4_ESM.docx]

Additional file 4. Results of the sensitivity analyses making the extreme assumption that all advanced CRCs that could not be stratified by UICC stage III vs. IV based on cancer registry (ZfKD) data were all UICC stage IV (see also Additional file 2)

1. Age-standardized incidence rates (ASIRs) of advanced CRCs with affected lymph nodes only (UICC III): Comparison between GePaRD and ZfKD
2. Age-standardized incidence rates (ASIRs) of advanced CRCs with distant metastases (UICC IV): Comparison between GePaRD and ZfKD
