# Supplementary material for: Incidence of advanced colorectal cancer in Germany: comparing claims data and cancer registry data
Source: BMC Med Res Methodol. 2019 Jul 8;19:142. doi: 10.1186/s12874-019-0784-y (PMC6615087; doi:10.1186/s12874-019-0784-y)
Supplement: Supplementary file 5 — Sensitivity analyses on age-standardized incidence rates (ASIRs) of advanced CRCs with affected lymph nodes only estimated based on claims data (GePaRD): Comparison of different periods used for the consideration of C77 codes after cohort entry. (DOCX 16 kb) [file 12874_2019_784_MOESM5_ESM.docx]

Sensitivity analyses on age-standardized incidence rates (ASIRs) of advanced CRCs with affected lymph nodes only estimated based on claims data (GePaRD): Comparison of different periods used for the consideration of C77 codes after cohort entry
